# Biogenesis and regulatory hierarchy of phased small interfering RNAs in plants

**DOI:** 10.1111/pbi.12882

**Published:** 2018-02-23

**Authors:** Pingchuan Deng, Sajid Muhammad, Min Cao, Liang Wu

**Affiliations:** ^1^ Department of Agronomy College of Agriculture and Biotechnology Zhejiang University Hangzhou China

**Keywords:** phased siRNA, *TAS*, one hit, NBS‐LRR, DNA methylation

## Abstract

Several varieties of small RNAs including microRNAs (miRNAs) and small interfering RNAs (siRNAs) are generated in plants to regulate development, genome stability and response to adverse environments. Phased siRNA (phasiRNA) is a type of secondary siRNA that is processed from a miRNA‐mediated cleavage of RNA transcripts, increasing silencing efficiency or simultaneously suppressing multiple target genes. *Trans*‐acting siRNAs (ta‐siRNAs) are a particular class of phasiRNA produced from noncoding transcripts that silence targets in *trans*. It was originally thought that ‘one‐hit’ and ‘two‐hit’ models were essential for processing distinct *TAS* precursors; however, a single hit event was recently shown to be sufficient at triggering all types of ta‐siRNAs. This review discusses the findings about biogenesis, targeting modes and regulatory networks of plant ta‐siRNAs. We also summarize recent advances in the generation of other phasiRNAs and their possible biological benefits to plants.

## Introduction

Small RNAs are short RNA molecules, 21–24 nucleotides (nt) in length, involved in the control of gene expression during development and environmental adaptation. Small RNAs in plants are categorized into two major classes according to their origin and biogenesis: microRNAs (miRNAs) and small interfering RNAs (siRNAs) (Axtell, 2013). Typically, miRNA precursors originating from stem–loop structures are processed by the RNase III enzyme Dicer‐like (DCL) into 20–24 nt fragments, and subsequently loaded onto Argonaute (AGO) proteins as part of the RNA‐induced silencing complexes (RISCs) to silence targets via nucleotide base‐pairing (Achkar *et al*., 2016; Megraw *et al*., 2016; Wu *et al*., 2009). While AGO1‐mediated RISCs promote 21‐nt miRNA suppression by mRNA digestion and translation inhibition, AGO4‐mediated epigenetic modification complexes guide 24‐nt miRNAs to target DNA methylation at the transcriptional level (Wu *et al*., 2010).

Common features of siRNAs and miRNAs are that they share the same processes of 3′ end modification by the RNA 2′‐O‐methyl‐transferase Hua enhancer 1 (HEN1) (Rogers and Chen, 2013). However, while siRNAs are enormously amplified by an RNA‐dependent RNA polymerase (RDR) after their generation, miRNAs are not (Matzke *et al*., 2015). siRNAs can be divided into three subclasses according to the differences in their origin and processing enzymes: heterochromatic siRNAs (hc‐siRNAs), natural antisense siRNAs (nat‐siRNAs) and phased siRNAs (phasiRNAs). hc‐siRNAs are 24 nt in length, transcribed by a plant‐specific RNA polymerase, PolIV, and processed via DCL3 from a genomic repeat or transposon region. After amplification involving RDR2 activity, hc‐siRNAs are recognized by AGO4 complexes and recruited by PolV with a long noncoding scaffold RNA to direct DNA methylation for the maintenance of genome stability and integrity (Matzke *et al*., 2015). nat‐siRNAs is a class of highly enriched small RNAs at the annealed pair regions of natural antisense transcripts, of which one gene is constitutively expressed while the other is inducible (Zhang *et al*., 2013).

**Figure 1:**
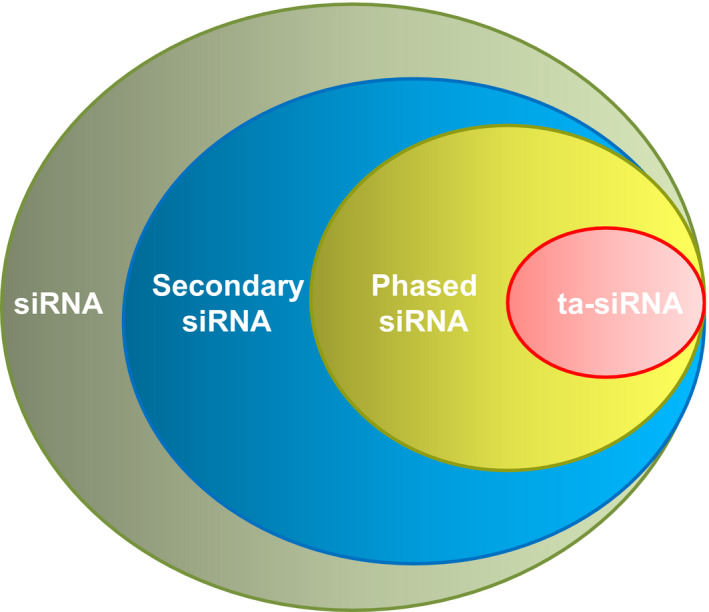
Diagram representing the scope of plant secondary siRNAs.

In animals, transport of nascent mRNAs from nucleus towards cytoplasm can be mediated by a multimeric protein complex, namely THO/TREX. If an RNA transcript in plants is exported by the THO/TREX complex and then becomes a template targeted by an miRNA, it can form numerous secondary siRNAs in a phased pattern relative to the miRNA cleavage site; therefore, these small RNAs are termed phasiRNAs (Fei *et al*., 2013). Furthermore, when a noncoding transcript is sliced by a miRNA, it generates a subset of phasiRNAs known as *trans*‐acting siRNAs (ta‐siRNAs) that can regulate target coding genes via mRNA cleavage in *trans* (Figure 1) (Fei *et al*., 2013). Nowadays, the recent development of sequencing approaches and resultant increase in the genomewide small RNA identification have enabled more secondary siRNAs to be characterized in a head‐to‐tail arrangement. Canonical phasiRNAs and ta‐siRNAs are readily distinguished, because when phasiRNAs are generated from noncoding transcripts and function outside of the local region, they are known as ta‐siRNAs.

To date, four families of *TAS* genes with eight loci have been discovered in the *Arabidopsis* genome. miR173 triggers three *TAS1* families and one *TAS2* family of ta‐siRNAs, miR390, initiates three *TAS3* families of ta‐siRNAs, and miR828 induces ta‐siRNAs at the *TAS4* locus (Rajagopalan *et al*., 2006). *TAS1*,* TAS2* and *TAS4* only exist in a limited number of plant species; however, *TAS3* ta‐siRNAs are conserved in thousands, from bryophytes to angiosperms (Xia *et al*., 2017). Compared with ta‐siRNAs, canonical phasiRNAs are less conserved (Xia *et al*., 2015a). In this review, we will mainly discuss the production of ta‐siRNAs and phasiRNAs and describe that how they behave in regulatory cascades rather than other small RNA actions, which have been extensively described earlier (D'Ario *et al*., 2017; Li *et al*., 2017).

## The biogenesis of phasiRNAs and ta‐siRNAs

**Figure 2:**
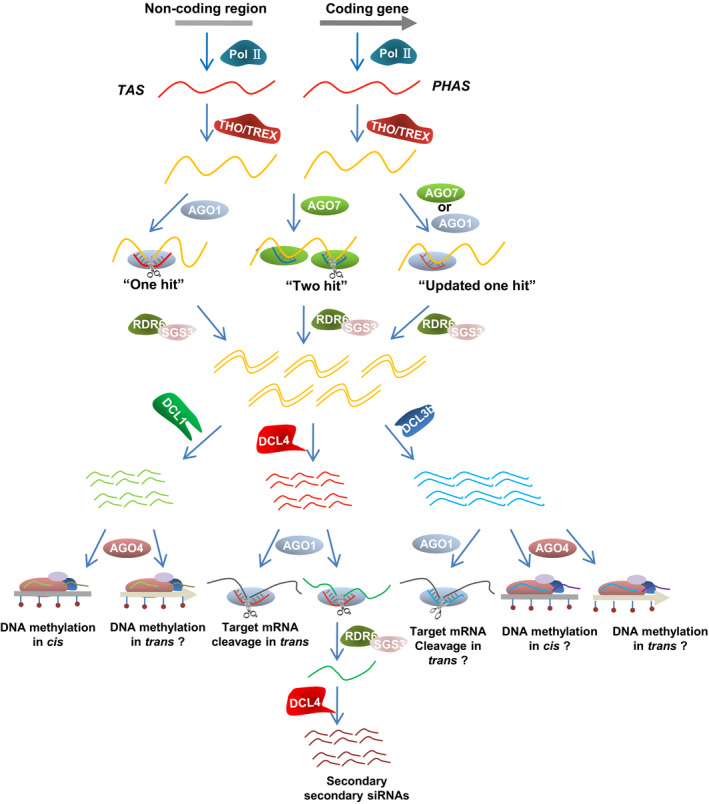
Biogenesis and activity hierarchy of ta‐siRNAs and phasiRNAs. The ta‐siRNA and phasiRNA precursors are transcribed by Pol II from noncoding loci and protein‐encoding genes, respectively. They are subsequently exported to the ARGONAUTE catalytic centre for miRNA‐mediated cleavage after trafficked by the THO/TREX complex. If the cleavable miRNA is 22 nt long or contains a bulge structure with miRNA*, it fits the ‘one‐hit’ model, in which the miRNA has a single target site and slices the target via AGO1. If the cleavable miRNA is a 21‐nt canonical miRNA, it fits the ‘two‐hit’ model, in which the miRNA has two target sites, but only one can be cleaved even though both interact with the miRNA in AGO7. An alternative ‘updated one‐hit’ model has recently been described, in which an interaction between the miRNA and the target sequence is sufficient to induce the production of secondary siRNA by AGO1 or AGO7, even in the absence of cleavage events. Fragments produced by the miRNA‐AGO complex are then protected and amplified by RDR6 and SGS3 to form double‐stranded RNAs, which are subsequently processed by DCL‐like proteins. Processing by DCL4 results in the generation of 21‐nt siRNAs that are recruited by the AGO1 complex to mediate the cleavage of target mRNA or induce the production of secondary siRNAs. Processing by DCL1 also leads to the generation of 21‐nt siRNAs, but these are incorporated into AGO4 clade proteins to mediate the methylation at their own DNA loci. Processing by DCL3 family proteins (e.g. DCL3b) results in the production of 24‐nt siRNAs that may associate with AGO1 to cleave target sequences or with AGO4 for DNA methylation. Whether *trans‐*active ta‐siRNAs and phasiRNAs can guide DNA methylation remains unknown.

Generally, miRNAs repress targets either by direct digestion or by translation inhibition in a homology‐dependent manner. However, when miRNAs of a particular length, structure or effector features cleave RNA molecules, they can generate small RNA precursors (Fei *et al*., 2013). These are amplified into double‐stranded RNAs (dsRNAs) by RDR6 with the binding factor SUPPRESSOR OF GENE SILENCING3 (SGS3). The dsRNAs are subsequently processed by DCL4 and DOUBLE‐STRANDED RNA BINDING FACTOR4 (DRB4) into 21‐nt secondary siRNAs in a ‘head‐to‐tail’ phased pattern (Figure 2). While the importance of RDR6 and DCL4 is largely known in the phasiRNA biogenesis pathway, the molecular mechanism by which SGS3 and DRB4 process phasiRNA is unclear, even though a dramatic decrease in phasiRNAs is observed in their respective mutants (Yoshikawa *et al*., 2013). These 21‐nt phasiRNAs are mainly deployed into AGO1‐mediated RISCs, and then function against other mRNAs. A class of 24‐nt phasiRNAs have been recently discovered during the reproductive stage in rice, and these small RNAs are processed by a DCL3 protein, DCL3b, rather than DCL4 for their biogenesis, even if their precursor dsRNAs are amplified by the SGS3 and RDR6 module as canonical 21‐nt phasiRNAs (Figure 2) (Johnson *et al*., 2009; Komiya, 2017; Song *et al*., 2012a,b). Whether these 24‐nt phasiRNAs are distributed into AGO4 complexes for transcription silencing or AGO1 effectors for mRNA degradation still remains elusive.

miRNA hijack action against long noncoding RNAs can lead to the production of ta‐siRNAs. The biogenesis model of ta‐siRNAs is a good example of how plants produce phasiRNAs. Thus far, four distinct classes of ta‐siRNAs have been characterized in *Arabidopsis*, which can be interpreted by two different mechanisms: ‘one hit’ and ‘two hit’.

In the ‘one‐hit’ model, a single miRNA guides target RNA transcript digestion through AGO1 RISCs, resulting in the generation of ta‐siRNAs 3′ downstream of the miRNA cleavage site (Figure 2) (Felippes and Weigel, 2009; Montgomery *et al*., 2008b). PhasiRNAs cannot triggered by all miRNAs, but only by those with 22 nt in length or a bulge structure in the miRNA and miRNA* duplex (Chen *et al*., 2010; Cuperus *et al*., 2010; Manavella *et al*., 2012). Because the generation of 22‐nt miRNAs usually results from an asymmetric duplex in the miRNA precursor, it is assumed that 22‐nt miRNAs or those with unpaired nucleotides are the same cause for triggering secondary siRNAs (Manavella *et al*., 2012). Intriguingly, although miR171 is a canonical 21‐nt miRNA with no asymmetric structure, it was recently shown to give rise to phasiRNAs in a *hen1* background (Zhai *et al*., 2013). This may be because of the presence of an additional nucleotide at the miR171 3′ terminal end in the *hen1* mutant, where small RNAs are increased uridylation (Zhai *et al*., 2013). This observation further suggests that miRNA with 22 nt in length is indeed important for phasiRNA biogenesis. Nonetheless, some exceptions of miRNAs also exist in the ‘one‐hit’ model, for instance, miR824, a 21‐nt miRNA and with no bulge structure between miRNA and miRNA*, can be loaded onto AGO1 to generate ta‐siRNAs at *TAS4* loci, although the mechanistic detail is still elusive (Rajagopalan *et al*., 2006). Taken simply, ‘one hit’ refers to the generation of phasiRNAs by an miRNA‐mediated cleavage event.

Compared with *TAS1* and *TAS2* ta‐siRNAs and most phasiRNAs, which are triggered by 22‐nt miRNAs through ‘one‐hit’ process, ta‐siRNAs from *TAS3* loci in *Arabidopsis* are initiated by the 21‐nt miR390 in a ‘two‐hit’ mechanism (Figure 2) (Axtell *et al*., 2006). Under this process, all three *TAS3* loci have two miR390 target sites, but only the 3′ proximal site of *TAS3* can be cleaved while the 5′ site loses its cleavage ability in *Arabidopsis* because it has fewer nucleotides that match miR390 (Montgomery *et al*., 2008a). miR390 3′ targeting seems not to be essential for *TAS3* ta‐siRNA biogenesis because phased siRNAs can still be generated if other miRNAs replace miR390 at the *TAS3* 3′ proximal site, as long as cleavage takes place (Montgomery *et al*., 2008a). By contrast, the *TAS3 5*′ region is indispensable for triggering ta‐siRNAs because a change in this miR390 binding site to an miRNA entirely blocks secondary siRNA generation (Montgomery *et al*., 2008a).

Another feature of the ‘two‐hit’ process is that miR390 must be recruited into AGO7 rather than an alternative AGO at the 5′ miR390 targeting site to trigger *TAS3* ta‐siRNAs (Figure 2) (Axtell *et al*., 2006; Montgomery *et al*., 2008a). The AGO7–miR390 complex has been proposed to operate through a ‘stable’ association, which is required for subsequent routing of *TAS3a* transcripts (Endo *et al*., 2013; Montgomery *et al*., 2008a). This may generate an intermediate aberrant poly(A)‐less substrate that facilitates the amplification by RDR6 (Baeg *et al*., 2017). Although miR390/AGO7 RISCs appear to have an essential noncleavable interaction at the 5′ site for *TAS3* ta‐siRNA biogenesis in *Arabidopsis*, this is not consistent across all plant species because both miR390 sites are cleavable in pine and moss (Axtell *et al*., 2006). Moreover, both cleavable and noncleavable configurations of miR390 and *TAS3* transcripts have been identified among distinct *TAS3* paralogs in spruce, implying that the machinery of the miR390–AGO7 module in *TAS3* transcripts of the ‘two‐hit’ model differs between different plant species (Xia *et al*., 2015a). It will be of interest to understand why distinct mechanisms occur at miR390 and *TAS3* 5′ binding sites.

Using a variety of artificial ta‐siRNA transgenic *Arabidopsis* lines that silence SULPHUR, an ideal system to observe the bleaching phenotype, de Felippes *et al*. recently showed that secondary siRNAs are still generated when the 5′ proximal miR390 target site of *TAS3* is mutated to allow cleavage (de Felippes *et al*., 2017). This mimics what occurs in moss, whereby two cleavable events at both the 5′ and 3′ ends impair the phasing pattern of one cleavage event in the ‘two‐hit’ configuration. Furthermore, a single slicing process at the *TAS3* 5′ proximal region by miR390 was found to be sufficient to trigger ta‐siRNA production, even if no cleavage occurs at the 3′ miR390 targeting region (de Felippes *et al*., 2017). This suggests that *TAS3* ta‐siRNA biogenesis is independent of miR390 cleavage at the 3′ end. Importantly, they showed that the generation of ta‐siRNAs could be achieved by a noncleavable interaction of miR390 and the TAS3 5′ target site (de Felippes *et al*., 2017). Although unexpectedly, triggering ta‐siRNA by an miRNA‐mediated noncleavage event is believable because ta‐siRNAs from *TAS1* in *Arabidopsis* can also be initiated by miR173 in a cleavage‐independent manner (de Felippes *et al*., 2017). Therefore, the ‘one‐hit’ model process appears to be applicable to all secondary siRNA syntheses. However, specificities on how ta‐siRNAs are generated from *TAS1* and *TAS3* loci still exist; for instance, the former requires 22‐nt miRNAs and AGO1‐mediated RISCs, whereas the latter relies on 21‐nt miRNAs and AGO7 activity. Although ‘one‐hit’ action at TAS3 is sufficient to generate ta‐siRNAs, the quantity and accuracy are lower than that achieved by ‘two‐hit’ action, suggesting that the ‘two‐hit’ model has evolved to improve the efficiency of secondary siRNA identity (de Felippes *et al*., 2017). Thus, secondary sRNA can be effectively triggered via a noncleavable miRNA interaction, but the absence of cleavage causes suboptimal phasing, possibly due to ill‐defined siRNA processing ends available for DCL4 action.

After processing by miRNA, the poly(A)‐less mRNA or noncoding RNA may function as a substrate for RDR6 to synthesize a complementary strand, and successively amplify the template of secondary siRNAs. However, it remains unclear that why the precursors of ta‐siRNAs and other phasiRNAs are specifically amplified by RDR6 rather than other RDR proteins. It is possible that RDR6 discrimination of secondary siRNA precursors is assisted by the plant‐specific coiled‐coiled protein SGS3, which has been proposed to play a role in the stabilization of primary siRNAs and cleavage products (Yoshikawa *et al*., 2005, 2013).

Intriguingly, some *TAS* primary transcripts, such as *TAS2*, has been observed with several short open reading frames, which can be translated and associate with *TAS* transcript fragments in polysome fractions (Yoshikawa *et al*., 2016). These form large complexes including SGS3, miRNA‐programmed RISCs and ribosomes (Yoshikawa *et al*., 2016). While it is uncertain that how these complexes are formed, modified and stabilized, they can affect the RDR6 amplification efficiency and resultant ta‐siRNA accumulations.

A recent finding shows that miRNAs and ta‐siRNA precursors are associated with membrane‐bound polysomes at the endoplasmic reticulum, where they can be recruited by AGO1 to exert their endonuclease activities (Li *et al*., 2016). In an AGO1 loss‐of‐function mutant, the amount of ta‐siRNAs and phasiRNAs was dramatically reduced because the triggering of miRNAs did not correctly associate with the membranes (Li *et al*., 2016). The location of the AGO complex is not important only, but AGO1 slicer activity is also required. Plants with defective AGO1 slicer activity cannot produce secondary siRNAs in a phased pattern at *TAS* loci, although their abundance is not obviously reduced (Arribas‐Hernandez *et al*., 2016). This finding is consistent with the ‘update‐one‐hit’ model that the interaction between miRNA and target mRNA rather than the cleavage is essential for secondary sRNA formation at *TAS* loci, even though the cleavage event seems to be crucial for their phasing pattern.

Once generated, long noncoding transcripts are often sliced into small RNAs by DCL proteins. DCL4 is the major DCL protein that recognizes transcripts produced by RDR6 and dices them into 21‐nt fragments in a phased manner (Yoshikawa *et al*., 2005). DCL2 and DCL3 may also be involved in ta‐siRNA and phasiRNA enhancement, because they can process sRNAs similarly when DCL4 is compromised (Gasciolli *et al*., 2005; Henderson *et al*., 2006).

After processing by DCL proteins, ta‐siRNAs and phasiRNAs are transported into the cytoplasm and sorted into different AGO complexes based on their 5′ terminal nucleotide (Mi *et al*., 2008). Although most ta‐siRNAs enter into AGO2 in *Arabidopsis* because of their 5′ adenine residue, their roles with AGO2 are not clear (Mi *et al*., 2008; Montgomery *et al*., 2008a). By contrast, although only a small proportion of ta‐siRNAs and phasiRNAs are loaded into AGO1, their biological importance is much obvious. Most phasiRNAs slice their target mRNAs in *cis*, while ta‐siRNAs cleave their target transcripts in *trans* (Fei *et al*., 2013). It is unclear whether ta‐siRNA and phasiRNA can repress targets via translation repression like miRNAs so far, but ta‐siRNAs are known with capacity to direct DNA methylation like hc‐siRNAs (Wu *et al*., 2012). The biological relevance of ta‐siRNA‐guided DNA methylation is still vague, because *TAS* expression is unchanged in the *ago4* mutant, which shows reduced methylation at *TAS* loci, and no ta‐siRNA target genes have been found to be methylated in *trans* yet (Wu, 2013; Wu *et al*., 2012).

Intestinally, the methylation levels not reduced in *PolIV*,* DCL3* and *RDR2* knockout mutants, suggesting the involvement of 21‐nt rather than 24‐nt hc‐siRNAs in *TAS* DNA methylation (Wu *et al*., 2012). Another unexpected observation is that ta‐siRNA‐mediated methylation is not compromised by the loss of DCL4 activity, even if most ta‐siRNAs are absent in the *dcl4* mutant, indicating that secondary siRNAs generated by DCL4 are not required for *TAS* loci methylation (Wu *et al*., 2012). Additionally, the methylation is consistent at the *TAS3a* locus in the *dcl2/3/4* triple mutant, indicating that small RNAs generated by DCL1 are responsible for this process (Wu *et al*., 2012). Meanwhile, DNA methylation guided by ta‐siRNAs is inhibited in the *ago4* and *polV* mutant, reminiscent of hc‐siRNA‐directed DNA methylation, suggesting a similar downstream pathway of DNA methylation by ta‐siRNAs and hc‐siRNAs (Wu *et al*., 2012). Based on these observations, two possible functioning pathways involving ta‐siRNA have been proposed. In the first, ta‐siRNA precursors are amplified by RDR6 and processed by DCL4 and then recruited into AGO1 to guide target mRNA cleavage (Figure 2). Alternatively, ta‐siRNA precursors may be directly processed by DCL1 to form small RNAs that are hierarchically loaded into AGO4 to mediate DNA methylation, but it is still unknown whether these *TAS* sRNAs are expressed in a phasing pattern (Figure 2). It is interesting to know in the future how DCL4 and DCL1 cooperate in ta‐siRNA biogenesis for differential action pathways.

## Regulatory cascade of ta‐siRNAs, phasiRNAs and their targets

### ta‐siRNAs and targets

While miRNAs can regulate diverse targets, ta‐siRNAs and phasiRNAs often target gene families. To date, four *TAS* families have been characterized in *Arabidopsis thaliana*, and the physiological functions of three of them have been described.

Both *TAS1* and *TAS2* are targeted by miR173. They regulate a gene family encoding the pentatricopeptide repeat (PPR) protein, which are widespread in dozens of plant species (Chen *et al*., 2007; Felippes and Weigel, 2009). Since the biological relevance of *TAS1*,*TAS2* and their *PPR* targets has not been revealed, it is unclear why such PPR‐encoding mRNAs need to be sliced by *TAS1* and *TAS2* ta‐siRNAs. However, the fact that *TAS1* mediates plant thermotolerance through regulation of several heat stress transcription factors has been validated recently (Li *et al*., 2014a). Exposure of plants to heat shock quickly represses *TAS1* ta‐siRNAs, resulting in an increase in *HEATINDUCED TAS1 TARGET1* (*HTT1*) and *HTT2* for heat tolerance enhancement (Li *et al*., 2014a).

Almost all plant developmental processes can be affected by auxin, an endogenous plant hormone. The auxin signalling pathway is eventually integrated to regulate DNA‐binding auxin transcription response factors (ARFs). The plant ARF family members can be targeted by miRNAs and *TAS3* ta‐siRNAs (Yamamuro *et al*., 2016). The ta‐siRNA‐ARF module is one of the most conserved plant small RNA‐target regulatory circuits and has been observed from simple organisms, including liverworts and ferns, to monocots and eudicots (Xia *et al*., 2017). Moreover, the initial 5′ adenosine of miR390 and the central region of the miR390/miR390* duplex have been found to be conserved and responsible for the interaction with AGO7 (Endo *et al*., 2013). This interaction occurs late during the specification of seed plants, which may reflect the complexity of the evolutionary path of the miR390‐*TAS3*‐*ARF* pathway in land plants. Another two relatively highly conserved regions of the miR390 precursors locate in either 5′ or 3′ lower stem of the miR390 hairpin structure, but their biological significance is still an enigma (Xia *et al*., 2017). Additionally, the number of *TAS3* loci is species dependent, being able to range from two to hundreds (Xia *et al*., 2015a, 2017). This diversity is suggestive of a long evolutionary history for the miR390‐*TAS3*‐*ARF* cascade in distinct land plant lineages.

The expressions of at least two *ARF* genes are consistently regulated by *TAS3* ta‐siRNAs in different plant species (Xia *et al*., 2017). Interestingly, the variability in the nucleotide sequence of the *TAS3* ta‐siRNA target sites in *ARF* genes is much lower than that of other positions, even if they are encoding conserved protein structure domains (Xia *et al*., 2017). This suggests that the *TAS3* ta‐siRNA target site is under a higher selective force than the conserved protein‐encoding regions. It is unclear why plants evolved such a complex system. Perhaps this regulatory mechanism could be more accurate than regulation via protein‐encoding domains over a long‐term evolutionary history.

The critical functions of the ta‐siRNA‐ARF module is to modulate leaf morphology, developmental transitions, flower and root architecture, embryo development, responses to biotic and abiotic stresses, and phytohormone crosstalk and these functions were characterized in *A. thaliana* several years ago (Table 1) (Adenot *et al*., 2006; Fahlgren *et al*., 2006; Marin *et al*., 2010; Matsui *et al*., 2014; Xu *et al*., 2007; Yoon *et al*., 2010; Yoshikawa *et al*., 2005). Recent studies have revealed that this module is also essential in adaptation to extreme environments via auxin networks in several other plant species (e.g. *Medicago truncatula*,* Lotus japonicus*,* Zea mays*,* Dimocarpus longan Lour*., and *Pyrus serotina*) (Bai *et al*., 2016; Dotto *et al*., 2014; Li *et al*., 2014b; Lin *et al*., 2015; Zhou *et al*., 2013). Particularly, the ta‐siRNA‐ARF module in the moss *Physcomitrella patens* contributes to auxin and nitrogen sensitivities, and this implies a cooption of regulatory networks by ta‐siRNAs and targets during the evolution of lower plants (Xia *et al*., 2016). Intriguingly, *TAS3* ta‐siRNAs are variable and have been indentified more potential targets other than ARFs in *Bruguieragy mnorrhiza* (Wen *et al*., 2016), suggesting an important role of ta‐siRNA and their regulatory networks in plant stress adaptations. Consistent with these implications, several AP2 transcription factors can be targeted by *TAS3* ta‐siRNAs in bryophytes (Axtell *et al*., 2007; Xia *et al*., 2017).

**Table 1:** Known plant ta‐siRNA and phasiRNA loci

Precursor name	Biogenesis model	Initiate miRNA name and length	Biological relevance	Family of plants	References
TAS1	Noncoding	miR173 22 nt	Heat tolerance	Eudicots	Li *et al*. (2014a)
TAS2	Noncoding	miR173 22 nt	Unknown	Eudicots	Yoshikawa *et al*. (2016)
TAS3	Noncoding	miR390 21 nt	Auxin signalling	Land plants	Xia *et al*. (2017)
TAS4	Noncoding	miR828 22 nt	Trichome development	Arabidopsis	Guan *et al*. (2014)
TAS5	Coding	miR482 22 nt	Disease resistance	Solanaceae	Li *et al*. (2012)
TAS6	Noncoding	miR156 21 nt miR529 21 nt	Targets a zinc finger protein; Unknown	Moss	Arif *et al*. (2012)
TAS7	Noncoding	vvimiRNA828a 22 nt vviTAS7‐primoRNA2 21 nt	Unknown	Grapevine	Zhang *et al*. (2012)
TAS8	Noncoding	vviTAS8‐primoRNA1 21 nt vviTAS8‐primoRNAA2 21 nt	Unknown	Grapevine	Zhang *et al*. (2012)
vviTAS9	Noncoding	vviTAS9‐primoRNA 21 nt	Unknown	Grapevine	Zhang *et al*. (2012)
vviTAS10	Noncoding	vviTAS9‐primoRNA1,2,5 22 nt vviTAS9‐primoRNA3,4,6 22 nt	Unknown	Grapevine	Zhang *et al*. (2012)
Sly‐TAS9	Noncoding	miR9470‐3p 22 nt	Chilling response	Solanaceae	Zuo *et al*. (2017)
Sly‐TAS10	Noncoding	miR00093 21 nt miR00105 21 nt	Chilling response	Solanaceae	Zuo *et al*. (2017)
Unnamed	Noncoding	miR2118 22 nt	Reproduction development	Rice and maize	Fan *et al*. (2016)
Unnamed	Noncoding	miR2275 22 nt	Reproduction development	Rice and maize	Zhai *et al*. (2015)
Unnamed	Noncoding	miR4392 22 nt	Reproduction development	Soybean	Arikit *et al*. (2014)
Cs1g09600 Cs1g09635	Noncoding	miR3954 22 nt	Flowering time	Citrus	Liu *et al*. (2017)
NBS‐LRR	Coding	miR1507 22 nt miR2109 22 nt miR2118 22 nt	Disease resistance	Medicago	Cakir *et al*. (2016)
NBS‐LRR	Coding	miR482 22 nt miR1507 22 nt miR1510 22 nt	Nodule development	Soybean	Zhai *et al*. (2011)
NBS‐LRR	Coding	miR472 22 nt miR482 22 nt	Disease resistance	Citrus	Wu *et al*. (2015)
Mla1	Coding	miR9863 22 nt	Disease resistance	Barley	Liu *et al*. (2014)
Ca^2+^‐ATPase	Coding	MiR4376 22 nt	Reproductive growth	Solanaceae	Wang *et al*. (2011)
MYB	Coding	miR828 22 nt miR858 22 nt	Secondary metabolism, seed development and fibre growth	Apple, soybean and cotton	Xia *et al*. (2012)
F‐box	Coding	MiRFBX 22 nt	Shape fruit	Strawberry	Xia *et al*. (2015a)
*TIR/AFB*	Coding	miR393 22 nt	Unknown	Arabidopsis	Wong *et al*. (2014)
NAC	Coding	miR3954 22 nt	Flowering time	Citrus and litchi	Liu *et al*. (2017)
ARF	Coding	miR167 22 nt	Unknown	Litchi	Ma *et al*. (2017)
PPR	Coding	miR7122 22 nt	Unknown	Solanaceae	Xia *et al*. (2013)
SGS3	Coding	miR2118 22 nt	Small RNA biogenesis	Soya bean	Zhai *et al*. (2011)
DCL2	Coding	miR1507 22 nt miR1515 22 nt	Small RNA biogenesis	Legumes	Xia *et al*. (2012)

Provided only one most recent and relevant reference for each TAS and PHAS loci due to the space limitation here.

In *A. thaliana*, a noncoding transcript generated from the *TAS4* locus between the *MYB* and *PROTEIN PHOSPHATASE 2A SUBUNIT A2* (*PP2A*) genes is processed by miR828, resulting in an accumulation of 21‐nt ta‐siRNAs, which further target other *MYB* transcripts for degradation in response to sugars (Luo *et al*., 2012; Rajagopalan *et al*., 2006). In cotton, fibre development is regulated by two homoeologous *MYB* genes, and one of them is targeted by miR828 and miR858 to generate 21‐nt phasiRNAs. Interestingly, the regions targeted by miR828 are *TAS4* orthologs in *A. thaliana* and cotton (Guan *et al*., 2014). Thus, miR828 can target coding and noncoding transcripts and thereby generates *cis‐* and *trans* siRNAs and phasiRNAs in different species, respectively, and both of them can influence trichome development (Table 1).

TAS5 was firstly described in tomato. A putative miR482 homologous precursor (i.e. sly‐MIR482d) with a asymmetric bulge miRNA/miRNA* duplex was identified as targeting tomato *Bacterial Spot Disease Resistance Protein 4* (*Bs4*) mRNAs. Thus, secondary siRNAs can be induced at *Bs4*, resulting in a self‐regulated expression (Li *et al*., 2012). However, in a strict sense, these secondary siRNAs are phasiRNAs rather than *bona fide* ta‐siRNAs, because they are not generated from noncoding transcripts and are not functional in *trans*.

Moss is considered to carry three *TAS6* genes because some noncoding transcripts can give rise to secondary siRNAs following slicing by miR156 and miR529 (Arif *et al*., 2012; Cho *et al*., 2012). All three *TAS6* genes are adjacent to *TAS3* loci. Even though ta‐siRNAs generated from the *TAS6a* and *TAS6b* have variable sequences (Cho *et al*., 2012), they are likely to be the same ta‐siRNA family with the same ancestor, because both of their precursors can be sliced by miR156 and their loci are close to *TAS3* (Cho *et al*., 2012). Furthermore, *TAS6a* and *TAS3a* are sharing a single primary transcript, and only separated by a small central intron (Cho *et al*., 2012). Thus, it is reasonable that the actions of miR156 and miR390 may be linked in biogenesis of *TAS6a* and *TAS3a* ta‐siRNA in moss. This is also supported by a fact that tasiARF can accumulate in *MIM156* plants, in which miR156 and *TAS6* activity is blocked (Cho *et al*., 2012). It is interesting to explore more crosstalk of miRNAs in generation of different ta‐siRNA classes.

To date, *TAS7–TAS10* has been only predicted in grapevine (Table 1) (Zhang *et al*., 2012). However, because little is known about their initiator miRNA and how they are induced, it is unclear whether they are *bona fide* ta‐siRNAs and how they function in grapevine. An independent class of siRNAs designated *TAS9* and *TAS10* ta‐siRNAs has been reported in a tomato fruit small RNA data set, when plants are undergoing low temperature stresses, but how they are processed still remains unknown (Zuo *et al*., 2017). Further identification of *TAS7‐TAS10* homologs in other plant species may help to clarify their biogenesis and behaviours.

### *TAS*‐like siRNAs and targets

In addition to *TAS* transcripts, two other long noncoding RNAs as a substrate that produce secondary siRNAs by miRNAs have been elucidated in rice (Johnson *et al*., 2009). Previous research revealed that miR2118 and miR2275 are complementary with and slicing two noncoding transcripts, producing 21‐nt and 24‐nt secondary siRNAs, respectively (Song *et al*., 2012a). While miR2118‐induced phasiRNAs that are dependent on DCL4, those generated by miR2275 require DCL3b activity for biogenesis (Song *et al*., 2012a). Although it is unknown whether these secondary siRNAs are *trans‐* or *cis‐*acting to targets, they may have important regulatory roles affecting propagation since they preferentially expressed in reproductive tissues (Johnson *et al*., 2009).

Photoperiod‐sensitive male sterility (PSMS), a gene locus responsible for initiation of two‐line hybrid rice breeding has been recently discovered related to *TAS*‐like siRNAs (Ding *et al*., 2012; Fan *et al*., 2016; Zhou *et al*., 2012). The photopheriod‐sensitive genic male sterility 1 (Pms1) locus encodes a long noncoding RNA *PMS1T* that was preferentially expressed in young panicles and targeted by miR2118, giving rise to 21‐nt phasiRNAs. A single nucleotide polymorphism in PMS1T nearby the miR2118 recognition site has been found to affect rice fertility, because the accumulation of secondary siRNAs is altered accordingly (Fan *et al*., 2016). The targets and functioning modes of these phasiRNAs still remains unclear, but they have been shown incorporated into MEIOSIS ARRESTED AT LEPTOTENE1 (MEL1), a rice germ cell‐specific AGO protein, during meiosis (Komiya *et al*., 2014; Nonomura *et al*., 2007). MEL1 can associate with siRNAs from over 700 long noncoding RNAs, which are potential targets of miR2118 (Komiya *et al*., 2014).

In rice, MULTIPLE SPOROCYTES1 (MSP1) and its cofactor TDL1a directly influence the number of sporocytes and anther development (Zhang and Yang, 2014). When *MSP1* and *TDL1a* are mutated, the precursor and mature 24‐nt secondary siRNAs generated by miR2275 are almost completely disappeared (Fei *et al*., 2016), suggesting that MSP1 and TDL1a affect male gamete formation via regulation of miR2275‐produced *TAS*‐like siRNAs. It has been observed that the spatial and temporal expressions of miR2275‐produced *TAS*‐like siRNAs are overlapped with those of *OsAGO1d*,* OsAGO2b* and *OsAGO18*, suggesting that these phasiRNAs may be acting through these three AGO proteins (Fei *et al*., 2016). Furthermore, miR2218 and miR2275 were also recently suggested to be involved in the dynamic biogenesis of meiotic phasiRNAs in maize in a cell type‐dependent manner (Zhai *et al*., 2015). Therefore, a thorough characterization of the molecular basis and functions of ta‐siRNA‐like secondary siRNAs involving miR2218 and miR2275 in monocots may be helpful for establishing male sterile germplasm for hybrid crop breeding (Qin *et al*., 2014).

Another *TAS*‐like noncoding transcript, which is targeted by a 22‐nt miR4392, can generate secondary siRNAs that are predicted to cleave transposable elements in soybean (Arikit *et al*., 2014). Because they are preferentially accumulated in anthers, they may have a specific function related to soya bean propagation.

Consequently, it seems that miRNA‐mediated ta‐siRNA biogenesis is a general regulatory mechanism in control of reproductive development in diverse crops.

### Other phasiRNAs and targets

In addition to noncoding transcripts, miRNA‐triggered secondary siRNAs can be produced from protein‐coding gene families. To date, genes encoding nucleotide‐binding site leucine‐rich repeat (NBS‐LRR) proteins have been shown to be the largest gene family targeted by miRNAs to generate phasiRNAs. Three 22‐nt *M. truncatula* miRNAs, including miR2275, miR2109 and miR2118, were the first class of miRNAs determined to be responsible for these phasiRNAs (Table 1) (Zhai *et al*., 2011). In soya bean, another leguminous plant, multiple phasiRNA‐generating loci (*PHAS* loci) overlapping with the *NBS‐LRR* genes are targeted by miR482, miR1507 and miR1510 and are preferentially expressed in nodules (Zhai *et al*., 2011). This implies that a phasiRNA‐induced post‐transcriptional modification may be essential in developing nodules. Interestingly, although the miR2109 sequence is conserved in both soya bean and *M. truncatula*, it can only initiate phasiRNAs in *M. truncatula* (Zhai *et al*., 2011). The reason behind this is the difference in length of miR2109 as it is 21 nt in soya bean, but 22 nt in *M. truncatul*. Thus, the same miRNA differing in length can have different functions through distinct functioning mechanisms.

So far, phasiRNAs generated at NBS‐LRR‐conserved motifs have been detected in diverse plant species, including spruce, grapevine, poplar, cotton, A*rabidopsis* and citrus (Boccara *et al*., 2014; Kallman *et al*., 2013; Wu *et al*., 2015) suggesting that ancient small RNAs limited *NBS‐LRR* expressions in these species. However, this phenomenon has not been observed in rice, perhaps because of the specific variations in defence mechanisms in monocots (Fei *et al*., 2013).

As mentioned above, miR482 induces phasiRNAs and mediates tomato bacterial spot disease resistance (Table 1) (Shivaprasad *et al*., 2012). Likewise, excessive production of miR482e in potato plants compromises the NBS‐LRR protein activity, resulting in plants hypersensitive to *Verticillium dahliae* infection (Yang *et al*., 2015). Similarly, in barley, miR9863 mediates the cleavage of *Mla1* alleles, which encode NBS‐LRR protein receptors, and induces phasiRNAs underlying immunity against the powdery mildew fungus (Liu *et al*., 2014). These results suggest that when plants are attacked by pathogens, they would up‐regulate *NBS‐LRR* expressions by suppressing the miRNA‐mediated gene silencing pathway. The phasiRNA–NBS‐LRR protein regulatory module prevents the constitutive NBS‐LRR expressions, which is deleterious for plant growth.

*PPR* is another large gene family that can be targeted by miRNAs to trigger phasiRNAs expanding post‐transcriptional regulatory activities. In addition to being regulated by ta‐siRNAs, a subset of *PPR* transcripts can be directly targeted by miR7122, a 22‐nt miRNA, to initiate phasiRNA biogenesis (Xia *et al*., 2013). Intriguingly, in apple and grapevine, *PPR* phasiRNA production can be induced at noncoding RNA transcripts by miR7122, which is similar to *TAS*‐like secondary siRNAs (Xia *et al*., 2013). Furthermore, in soya bean and *M. truncatula*,* PPR* phasiRNA can be processed by a secondary siRNA cascade, which are produced by miR1509‐mediated cleavage (Xia *et al*., 2013). Thus, one, two and three layers of phasiRNA productions can give rise to an elaborate regulatory hierarchy for multiple *PPR* regulations. Additionally, miR4376,22‐nt miRNA regulates the expression of *ACA10*, an auto‐inhibited Ca^2+^‐ATPase gene through phasiRNAs to control tomato reproductive development (Wang *et al*., 2011). According to sequence similarities, miR7122 may have evolved from miR4376 (Xia *et al*., 2013). Meanwhile, the miR4376 and miR390 sequences are also similar, which is suggestive of an evolutionary relationship among miR390‐miR4376‐miR7122 (Xia *et al*., 2013). However, these three miRNA families regulate distinct genes to be involved in different biological processes; for instance, miR390 regulates *ARFs* via *TAS3*‐derived ta‐siRNAs and miR4376 mediates cleavage of Ca^2+^‐ATPase transcripts, while miR7122 regulates *PPR* expressions. These findings suggest that target sequence variations may cause evolution of miR390 to miR4376 and miR7122.

MYB transcription factor genes are the third richest source of miRNA‐mediated phasiRNAs. On the one hand, as mentioned above, *A. thaliana TAS4* noncoding RNA transcripts are targeted by miR828 to generate ta‐siRNAs that down‐regulate *MYB* genes (Allen *et al*., 2005; Rajagopalan *et al*., 2006). However, on the other hand, miR828 and miR858 in apple, soya bean and cotton can directly target some *MYB* motif transcripts to generate phasiRNAs that further prevent the accumulation of MYB transcription factors (Guan *et al*., 2014; Xia *et al*., 2012; Zhai *et al*., 2011). This process has been found with consequences for secondary metabolism, seed development and fibre growth in apple, soya bean and cotton plants, respectively (Guan *et al*., 2014; Xia *et al*., 2012).

*F‐box* is one of the largest gene families in plants, which encode the substrate‐discriminating subunits of the SCF (i.e. S‐phase kinase‐associated protein1–Cullin–F‐Box) ubiquitin ligases, and can affect numerous biological processes. One‐third of the strawberry *F‐box* genes have been illustrated to be targeted by a 22‐nt miRFBX cluster, resulting in the generation of a set of phasiRNAs (Table 1) (Xia *et al*., 2015b). The miRFBX7–F‐box–phasiRNA network has been observed only in wild diploid strawberry plants, suggesting that it can regulate strawberry‐specific processes (Xia *et al*., 2015b). Consistent with this notion, the miRFBX precursor transcripts are highly enriched in the cortex and pith tissues of the receptacle, a unique part of the strawberry flower that develops rapidly to produce the characteristic fruit shape (Xia *et al*., 2015b).

In addition to gene families, some low‐copy genes are also able to produce phasiRNAs when their transcripts are being cleaved by a 22‐nt miRNA. For example, the transcripts of *TRANSPORT INHIBITOR RESPONSE* (*TIR*) and *AUXIN SIGNALING F‐BOX* (*AFB*) genes are repressed by miR393, which mediates *A. thaliana* innate immunity through an enhancement of auxin response (Navarro *et al*., 2006). Interestingly, a couple of secondary siRNAs have recently been identified at *TIR/AFB* transcript sites cleaved by miR393, and these siRNAs are found very low in *dcl1*,* dcl4*,* rdr6*,* sgs3*,* ago1* and *mir393b* mutants (Si‐Ammour *et al*., 2011), implying that the processing of these siRNAs is dependent on a canonical phasiRNA biogenesis routine. In addition, two protein‐encoding gene transcripts were found to be sliced by these *TIR/AFB* secondary siRNAs in *trans*, suggesting that these phasiRNAs are functional, even though their involvement in auxin signalling is still unknown (Si‐Ammour *et al*., 2011). Similar as *TIR/AFB*, a limited number of *NAC* (*NAM, ATAF and CUC*) domain transcription factor transcripts are reported yielding phasiRNAs in citrus and litchi by miR3954, and their roles has been implicated in flowering regulation (Liu *et al*., 2017; Ma *et al*., 2017), further providing evidence that phasiRNAs from low‐copy genes are also important for plant development.

Plant genes encoding components of small RNA pathway can also be served as phasiRNA‐producing loci if the corresponding transcripts are cleaved by miRNAs. The *SGS3* orthologs in leguminous plants including soya bean and *M. truncatula* were predicted to be miR2118 targets (Zhai *et al*., 2011). Unexpectedly, *SGS3* phasiRNAs can be detected in soya bean, but not in *M. truncatula* (Fei *et al*., 2013). This may be because additional factors required for *SGS3* phasiRNA biogenesis exist in soya bean, but are lacking in *M. truncatula*. Unlike *SGS3* transcripts, which are targeted by the same miRNA, *DCL2* transcripts are targeted by distinct miRNAs in different leguminous species (Fei *et al*., 2013). For example, miR1507 and miR1515 can initiate the generation of *DCL2* phasiRNAs in *M. truncatula* and soya bean, respectively (Table 1) (Fei *et al*., 2013). Collectively, the production of phasiRNAs from *DCL2* and *SGS3* sequences suggests an important mechanism responsible for exquisite control of small RNA biogenesis and activities in legumes, reminiscent of feedback‐based regulation of *DCL1* and *AGO1* expression by miR162 and miR168 in *A. thaliana,* respectively.

A recent finding shows that Cuscuta, an obligate parasitic plant that absorbs water and nutrients from the host plants, accumulates high levels of 22‐nt miRNAs, which can target Arabidopsis and tobacco mRNAs and produce secondary siRNAs (Shahid *et al*., 2018). This is an efficient approach for Cuscuta to suppress gene expression and parasitize in hosts, suggesting that phasiRNA is also an important mechanism of trans‐species gene regulation. Considering the rapid increases in plant genome and transcriptome data, it is certain that more phasiRNAs will be characterized in near future. Nevertheless, illustration of phasiRNA biological significance should also be paid attention onward and speed up the pace.

## Conclusions and remarks

Over the last decade, ta‐siRNAs were first demonstrated to be involved in *A. thaliana* phase transitions (Peragine *et al*., 2004; Vazquez *et al*., 2004). Subsequently, additional ta‐siRNAs have been identified and the genetic requirements underlying their processing have been elucidated. Meanwhile, two models for ta‐siRNA biogenesis had been proposed, although it is still unclear if they can explain the generation of all secondary siRNAs. A special AGO7 complex required for *TAS3* ta‐siRNA biogenesis was discovered that made researchers interested in how miRNAs induce the generation of secondary siRNAs. One of the major breakthroughs involved the discovery of 22‐nt miRNAs essential for phasiRNA biogenesis, although the bulge structures of miRNA and miRNA* may also be required for this process. Since then, researchers have uncovered numerous phasiRNAs in diverse plants, suggesting a conserved biogenesis and related regulatory cascade among plant species.

Several issues regarding ta‐siRNAs and phasiRNAs need to be elucidated. For example, it is unclear how 22‐nt small RNAs generate secondary siRNAs. Perhaps, only 22‐nt small RNAs including miRNAs and siRNAs can link AGO1 with RDR6, SGS3 or other unidentified proteins at secondary siRNA precursors to precisely slice transcripts in a phased pattern. To address this issue, clarifying the crystal structures of the central components associated with 22‐nt small RNAs is necessary. Twenty two‐nt small RNAs are generally considered essential determinants of phasiRNA biogenesis, but with exceptions. For example, miR390, which is a canonical 21‐nt miRNA, can guide *TAS3* transcripts to ta‐siRNAs during a process mediated by AGO7 rather than other AGO‐mediated RISCs. Thus, future studies should investigate how AGO7 competes with and replaces AGO1 to interact with miR390 to induce the formation of *TAS3* ta‐siRNA. Furthermore, the interaction between specific miRNAs and ta‐siRNA precursors rather than a cleavage event is sufficient to initiate the production of phasiRNAs. This implies that phasiRNA biogenesis may be more complex than previously considered. Therefore, the components responsible for the interactions between miRNAs and phasiRNA precursors need to be isolated. Furthermore, only a limited number of gene families, low‐copy genes and noncoding RNAs have been shown processed into secondary siRNAs by miRNAs; thus, further study is required to reveal how miRNAs recognize specific targets for phasiRNA biogenesis. In addition to this, ta‐siRNAs are able to mediate DNA methylations at their own loci through an alternative RdDM pathway. However, this epigenetic process seems to be unrelated to the production of ta‐siRNAs and their precursors. Therefore, the effects of ta‐siRNA‐mediated DNA methylation and whether *trans‐*active ta‐siRNAs modify targets remain to be determined.

The CRISPR‐Cas9 system and gene silencing techniques are the two most efficient options for generating mutants for investigating gene functions. However, a loss‐of‐function mutant cannot be generated by gene editing if the mutation causes lethality. Moreover, the CRISPR‐cas9 approach is inappropriate for silencing a specific alternative splicing isoform, but alternatively, the application of an artificial miRNA is suitable (Qin *et al*., 2017; Wu *et al*., 2013). Thus, small RNA‐mediated gene silencing is still an important platform for reverse genetic studies on plant gene functions as well as crop trait improvement. A single *TAS* locus can be engineered to produce artificial ta‐siRNAs, which can increase the efficiency of one or multiple target genes silencing (Baykal *et al*., 2016; Carbonell *et al*., 2014). Artificial phasiRNAs are currently being used to rapidly generate plants that are resistant to viruses and viroids, suggesting ta‐siRNAs may be useful for improving important crops (Carbonell and Daros, 2017; Chen *et al*., 2016).

In conclusion, the discovery of plant ta‐siRNAs and phasiRNAs has contributed greatly to the advances that have been made in the research of RNA‐mediated gene silencing. Because ta‐siRNAs have also been predicted in small RNA data set of human brain (Liu *et al*., 2015), it is interesting to determine whether ta‐siRNAs and phasiRNAs are active beyond plant species.
